# Histogenous Hypoxia and Acid Retention in Schizophrenia: Changes in Venous Blood Gas Analysis and SOD in Acute and Stable Schizophrenia Patients

**DOI:** 10.3389/fpsyt.2021.792560

**Published:** 2021-12-06

**Authors:** Xingbing Huang, Qiu-Ling Lu, Xiu-Mei Zhu, Yi-Bin Zeng, Yun Liu, Hao-Ying Hu

**Affiliations:** ^1^Affiliated Brain Hospital of Guangzhou Medical University, Guangzhou, China; ^2^First Affiliated Hospital of Guangzhou Medical University, Guangzhou, China

**Keywords:** schizophrenia, SOD, oxidative stress, histogenous hypoxia, acid retention, venous blood gas, venous pH, PvO_2_

## Abstract

**Background:** Oxidative stress may play an important role in the pathogenesis of schizophrenia (SCH), and there is considerable indirect evidence that hypoxia is closely related to SCH, but direct evidence of hypoxia in SCH has never been found.

**Methods:**In this study, superoxide dismutase (SOD), venous blood gas, and Positive and Negative Syndrome Scale (PANSS) were examined in 40 SCH patients and compared with those of 40 healthy controls. The patients were treated with combination of atypical antipsychotics and Ditan Huayu Lishen decoction (a Chinese medicine decoction) and examined in the acute and stable period, respectively. Comparisons of indicators between two groups were performed using an independent-samples *t*-test, comparison of indicators between the acute and stable periods in the SCH group was performed using paired-samples *t*-test. Pearson correlation and multiple linear regression analyses were performed to investigate the relationships between the effect indicators.

**Results:** Higher venous pH, PvO_2_, and fasting blood glucose levels and lower SOD, lactic acid, and PvCO_2_ levels were found in SCH patients compared with the control group; SOD was negatively correlated with the general psychopathology subscale score (PANSS-G), and PvO_2_ levels were closely related to venous pH in SCH and related to PvCO_2_ in the control group. It was also found that SOD activity showed no significant difference in acute and stable period, whereas PvO_2_ showed a downward trend, and venous pH was decreased significantly after treatment. Both the venous pH and PvO_2_ were higher in patients with SCH than that in healthy controls.

**Conclusion:** It suggests that histogenous hypoxia and acid retention exist in relation to SCH, and there is an improvement of acid retention and a downward trend in histogenous hypoxia after combination treatment. Venous pH, PvO_2_, and PvCO_2_ are trait variables, but not state variables of SCH. The theory of histogenous hypoxia and acid retention can well explain the decrease in pH value and the increase in lactic acid in brain tissue of patients with SCH. Histogenous hypoxia and acid retention closely related to glucose metabolism. So they may play an important role in pathophysiology for SCH.

## Introduction

Schizophrenia (SCH) is a category of major mental health disorders of unknown etiology that encompasses significant abnormalities in cognitive, logical, emotional, behavioral, and other mental activities and leads to significant occupational and social function limitations (1). The lifetime prevalence of SCH was found to be 0.6% in a recent epidemiological survey of mental disorders in mainland China (2). Although genetic and environmental factors are thought to be involved in the pathogenesis of the disease, the cause of SCH remains unclear.

There is increasing evidence, however, that oxidative stress may play an important role in the pathogenesis of SCH (3). People with SCH have been found to have impaired antioxidant defenses and experience increased oxidative damage (4, 5). Severe oxidative stress can lead to decreasing antioxidant content and an increase in the production of free radicals, leading to cell damage and even cell death (6). According to the meta-analysis results, the level of activity of erythrocyte superoxide dismutase (SOD) was reduced in acuter elapse of psychosis, drug-naive first-episode psychosis, stable medicated outpatients, and chronic inpatients (7). Markers of free radical oxidation products prove the predominance of pro-oxidant processes in schizophrenia. The most reliable proof of predominance of pro-oxidant processes is the increase in thiobarbituric acid reactive substances in drug-naive first-episode psychosis, stable medicated outpatients, and chronic inpatients, which is confirmed by the results of meta-analysis (7, 8).

Prior research has also demonstrated that exposure of healthy humans to chronic intermittent hypoxia increased oxidative stress by overproduction of reactive oxygen species (ROS) (9), and intracellular ROS paradoxically increased under hypoxic conditions (10). There is an extensive literature of indirect evidence suggestive of a role for hypoxia in SCH pathophysiology. There is, for example, a strong association between fetal hypoxia and future development of SCH (11), increased expression of hypoxia-inducible factors in SCH postmortem prefrontal cortex (12), and a much higher proportion (55%) of genes associated with SCH being regulated by hypoxia and/or expressed in vasculature than would be expected by chance (13).

Although there is considerable indirect evidence that hypoxia is closely related to schizophrenia, direct evidence of hypoxia in SCH has never been found.

Abnormalities of oxidative stress are also known to exist in individuals with depression. In previous research, the current study's corresponding author confirmed the presence of histogenous hypoxia and an increased propensity for the accumulation of acidic metabolites in depressed patients by comparing samples of their venous blood gas (VBG) with those of healthy controls (14). The present research considers whether people with SCH also exhibit histogenous hypoxia and acid retention.

The purpose of the present study is to clarify whether there are abnormal oxidative stress linked to SCH and whether histogenous hypoxia and acid retention may be present. However, the effect of pharmacotherapy on these indicators remains unclear; furthermore, the link between them and the psychiatric symptoms of SCH remain unknown. To address these unknown aspects, we designed this study to compare the SOD and VBG analysis results in SCH patients with those of a healthy control sample group, as well as a self-controlled study to compare SOD and VBG results in acute and stable SCH patients.

## Materials and Methods

### Participants

The research participants were SCH patients and controls. The SCH patients were recruited from among inpatients attending the Department of Traditional Chinese Medicine in The Affiliated Brain Hospital of Guangzhou Medical University. The control group was recruited from among the staff members of the Department of Traditional Chinese Medicine and was matched approximately with the SCH patients by gender and age.

The inclusion criteria for the SCH group were, first, that the prospective participant's current symptoms met the diagnosis of SCH according to the *International Statistical Classification of Diseases and Related Health Problems* (code F20) (15). In other words, they are in the acute phase of schizophrenia; second, that they were between 18 and 60 years old. Exclusion criteria for the SCH group were as follows: (a) presence of fatty liver disease, hypertension, gout, renal failure, diabetes, or other medical diseases; (b) abuse of alcohol and/or psychoactive substances; (c) pregnant or lactating female patients; (d) patients confined to bed or a wheelchair; and (e) infection or alcohol-intake history within the previous week.

The inclusion criteria for the control group were as follows: (a) a good health record, featuring no physical illness or psychiatric disorder; (b) predominantly normal results from the most recent examination; (c) no sleeping disorders; and (d) no infection or alcohol-intake history within the previous week. Exclusion criteria for the control group were as follows: (a) presence of fatty liver disease, hypertension, gout, renal failure, diabetes, or other medical diseases; (b) abuse of alcohol and/or psychoactive substances; (c) pregnant or lactating female patients; (d) patients confined to bed or a wheelchair; and (e) infection or alcohol-intake history within the previous week.

### Clinical Interviews

Participants from both groups were invited to attend a clinical interview. The SCH patients were interviewed by an experienced psychiatrist to determine whether they met the screening criteria.

All participants were informed of the purpose, procedure, and possible benefits and risks of participation in this study. Participants who agreed to join the study were asked to sign informed consent forms and register their general data, encompassing the following: (a) background information, (b) demographic items, (c) previous history, and (d) medical history.

### Assessment of Symptom Severity

Participants in the SCH group were assessed by an experienced psychiatrist, who used the Positive and Negative Symptom Scale (PANSS) (16) to measure the positive, negative, and general symptoms of psychopathology. This evaluation tool is widely applied in China, and its reliability and validity have been verified; the internal consistency reliability (Cronbach α) was 0.87, whereas the internal consistency reliability of the five dimensions (e.g., positive symptoms, negative symptoms, depression, mania, and cognitive impairment) ranged from 0.74 to 0.90 (17). The analysis indicators incorporated scores from the PANSS subscale pertaining to positive symptoms (PANSS-P), the subscale measuring negative symptoms (PANSS-N), and the general psychopathology subscale (PANSS-G), as well as the total PANSS score (PANSS-T).

### Assessment of Biochemical Measures

Elbow vein fasting blood was drawn in the morning from each participant through several tubes. Indicators included VBG analysis, SOD, and general biochemical indicators (evaluated through lipid profile and kidney function).

A single-use arterial blood gas needle was used for the VBG analysis, with samples taken from the elbow vein and tested using a fully automatic blood gas analyzer within 15 min of the sample being collected. The analysis indicators included the venous pH value, the venous partial pressure of oxygen (PvO_2_), the venous partial pressure of carbon dioxide (PvCO_2_), lactic acid, potassium ions (K^+^), and fasting blood glucose (FBG).

SOD activity was determined with method of pyrogallol autoxidation in clinical laboratory of Affiliated Brain Hospital of Guangzhou Medical University.

### Intervening Measure

Control group is no longer special intervening measure. The SCH group was conducted with combination treatment of atypical antipsychotics (AASs) and Chinese medicine decoction in hospital. AAS included olanzapine, clozapine, risperidone, quetiapine, and aripiprazole with routine dosage in this study. The Chinese medicine decoction was named Ditan Huayu Lishen Decoction for purging phlegm, removing blood stasis, and tranquilizing the mind, which was composed of the following ingredients: fructus aurantii immaturus, *Pinellia, Poria, Curcuma longa* 15 g each, and *Bryozoatum* 20 g, bile *Arisaema* 10 g, *Fritillaria, Rhizoma acori graminei*, peach kernel, flowers *Carthami*, zedoary, *Hirudo, Eupolyphaga* 15 g each, *miltiorrhiza* 20 g, licorice 10 g, *Rheum officinale* 15 g (decoct later), and succinum 5 g (take drenched). The recipe is suitable for various mental diseases caused by phlegm and blood stasis (18). The compositions of the recipe were soaked in 800 ml water for 15 min and then boiled with high heat and simmered for half an hour. The filtrate was taken two times every day.

### Reassessment

The vein blood gas analysis, SOD, and PANSS were evaluated after 4 weeks' intervention in hospital in the SCH group.

### Statistical Analyses

All statistical analyses were performed using the PASW Statistics 20.0 software package. The measured data were presented with mean ± standard deviation (SD) following the data normality test. Enumeration data were presented with the constituent rate and ratio units. The test level was set to α = 0.05. The effect size (Cohen *d*) was calculated with web-based effect-size calculator in https://www.campbellcollaboration.org/.

#### Measurement Data

For normally distributed data, comparison between two groups was performed using an independent-samples *t*-test; comparison between before and after treatment in the SCH group was performed using paired-samples *t*-test. The relationship between two variables was determined through Pearson correlation analysis. The relationship between a dependent variable and multiple factors was determined using multiple linear regression analysis.

#### Enumeration Data

Comparison of constituent rates and ratios between two groups was carried out with a *χ*^2^ test or Fisher exact test.

## Results

### Characteristics of the Participants

There were 40 participants in the SCH group−25 males (62.5%), 15 females (37.5%)—and 40 in the control group−24 males (60.0%), 16 females (40.0%). There was no significant difference in gender balance between the two groups.

In the SCH group, there were four participants (10%) who were postmenopausal; two participants (5%) held religious beliefs, but 38 cases (95%) did not; four participants (10%) reported a history of smoking; two (5%) had experienced first-episode psychosis and 38 (95%) recurrent episodes; 23 (57.5%) were receiving treatment (e.g., olanzapine, clozapine, risperidone, quetiapine, aripiprazole), whereas 17 (42.5%) were not, and two cases had never been treated. The average withdrawal period from antipsychotic treatment was 9.71 months (1, 384). The course of the disease was 102.30 ± 99.93 months. The course of Western medicine treatment was 79.61 ± 95.07 months.

In the control group, three participants (7.5%) were postmenopausal; three (7.5%) held religious beliefs, and 37 (92.5%) did not; six participants (12%) reported a history of smoking. Thus, no significant differences were found between the two groups regarding the respective distribution of postmenopausal, religious beliefs, and smoking history data.

The two groups featured no significant differences in terms of participants' relative ages, heights, weights, and body mass indexes (BMIs), but there were differences regarding education levels: the years of education completed by participants in the SCH group were lower than those in the control group (Table 1).

**Table 1:** Participants' ages, education levels, heights, weights, and BMIs (mean ± SD).

**Variable**	**SCH group**	**Control group**	***t***	***P***
	***n* = 40**	***n* = 40**		
Age (years)	33.78 ± 11.62	32.88 ± 10.40	0.365	0.716
Education (years)	10.83 ± 3.49	15.08 ± 3.58	−5.383	0.001^*^
Height (cm)	166.08 ± 6.92	166.79 ± 9.58	−0.375	0.709
Weight (kg)	62.64 ± 11.00	62.65 ± 11.48	−0.004	0.997
BMI (kg/m^2^)	22.68 ± 3.54	22.43 ± 3.03	0.332	0.740

*Independent-samples t-test*.

* *p < 0.01*.

### Comparison in Baseline

**Figure 1:**
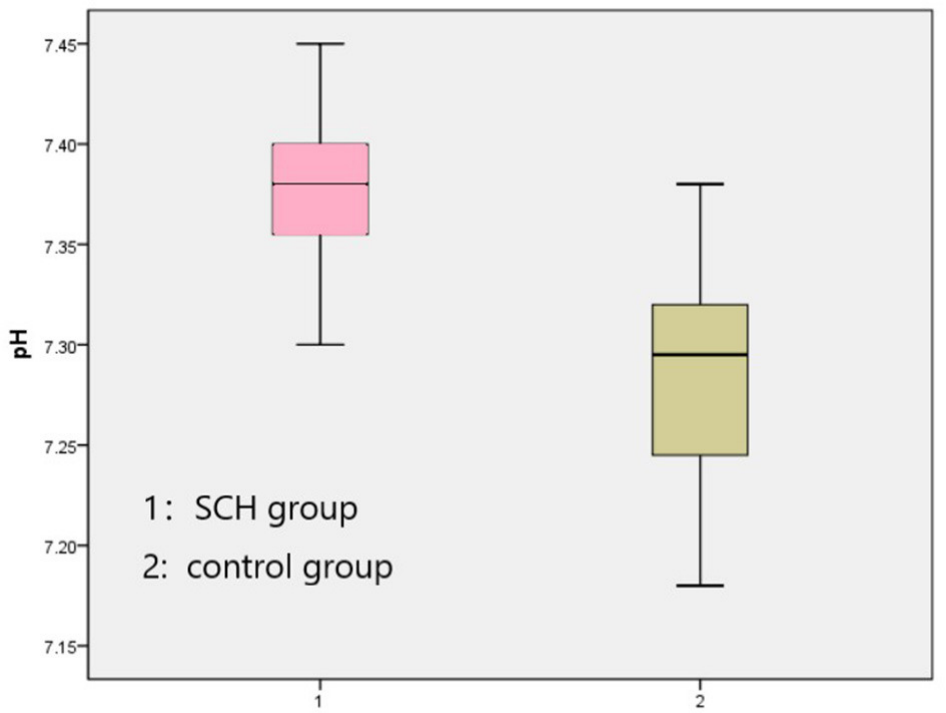
Comparison venous pH between two groups.

**Figure 2:**
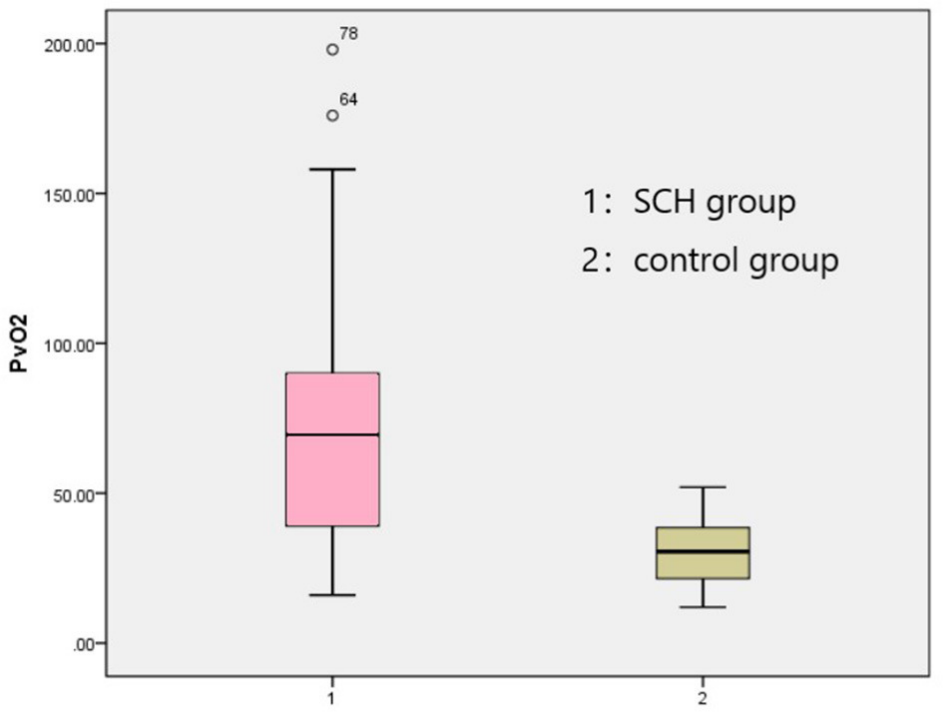
Comparison PvO_2_ between two groups.

**Figure 3:**
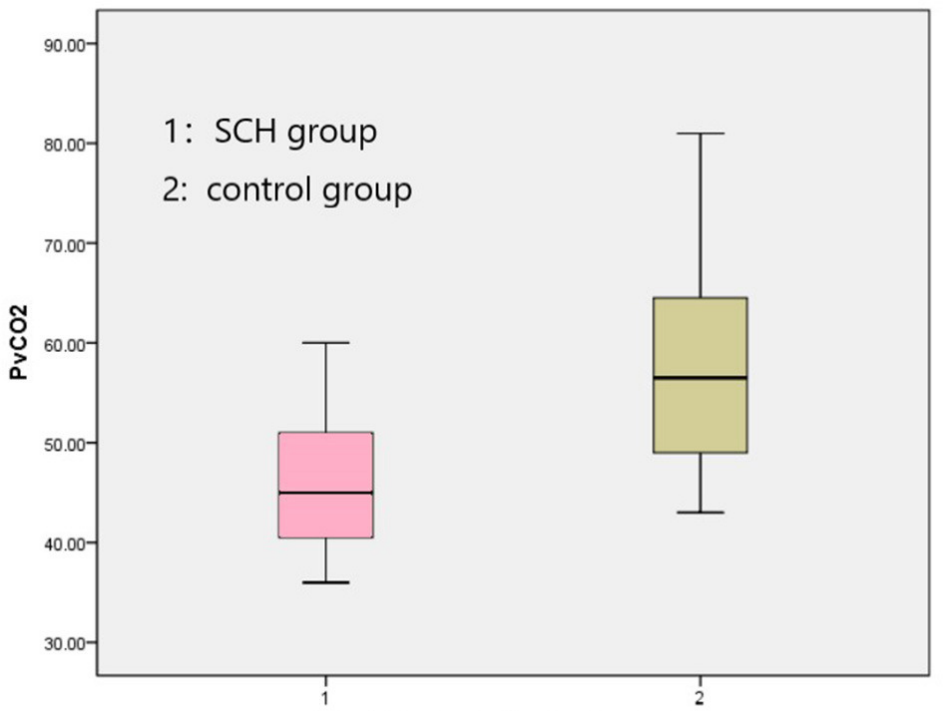
Comparison PvCO_2_ between two groups.

The venous pH, PvO_2_ values, and FBG in the SCH group were higher than those in the control group, whereas PvCO_2_ and lactic acid levels were lower, indicating statistical significance (*p* < 0.001 in all). There were no significant differences in K^+^ levels between the two groups. The SOD levels in the SCH group were lower than those of the control group, indicating statistical significance (*p* < 0.001). (Table 2; Figures 1–3).

**Table 2:** VBG analysis and SOD (*mean* ± SD).

**Variable**	**SCH group**	**Control group**	***t***	***P***	**Cohen *d***
	***n* = 40**	***n* = 40**			
Venous pH	7.375 ± 0.032	7.284 ± 0.051	9.493	<0.001^*^	2.138
PvO_2_ (mm Hg)	74.08 ± 43.42	30.00 ± 10.72	6.233	<0.001^*^	1.394
PvCO_2_ (mm Hg)	45.73 ± 6.53	58.33 ± 11.02	−6.219	<0.001^*^	−1.391
Lactic acid (mmol/L)	2.17 ± 1.09	3.55 ± 1.27	−5.214	<0.001^*^	−1.207
K^+^ (mmol/L)	3.67 ± 0.35	3.54 ± 0.25	1.839	0.070	0.427
FBG (mmol/L)	5.11 ± 0.88	4.05± 0.93	5.244	<0.001^*^	1.171
SOD (U/ml)	157.23 ± 31.95	196.38 ± 14.85	−7.027	<0.001^*^	−1.572

*Independent-samples t-test*.

* *p < 0.01*.

### Correlation Analyses

#### General Condition and the Effect Indicators

In order to clarify whether a participant's general condition (i.e., age, education, smoking history, height, weight, BMI, first episode, course of disease, course of treatment) had an impact on any of the effect indicators, correlation analysis between them was undertaken. It was found that SOD was positively correlated with BMI, and FBG was positively correlated with course of disease and course of treatment in the SCH group (Table 3), whereas age was positively correlated with venous pH and negatively correlated with PvCO_2_ and SOD, education was negatively correlated with K^+^, and height was positively correlated with PvCO_2_ in the control group (Table 4).

**Table 3:** Correlation analysis between the general situation and the effect indicators in the SCH group.

		**Age**	**Education**	**Smoking history**	**Height**	**Weight**	**BMI**	**First episode**	**Course of disease**	**In treatment**	**Course of treatment**
PANSS-P	*r*	−0.026	0.011	−0.036	−0.162	−0.208	−0.164	0.092	−0.074	0.092	−0.073
	*p*	0.873	0.947	0.827	0.323	0.204	0.320	0.572	0.649	0.573	0.654
PANSS-N	*r*	−0.010	−0.037	0.042	0.008	−0.047	−0.043	0.134	0.003	0.299	−0.078
	*p*	0.950	0.819	0.796	0.960	0.777	0.795	0.410	0.987	0.061	0.632
PANSS-G	*r*	−0.022	0.038	0.095	−0.129	−0.136	−0.086	0.084	−0.088	0.161	−0.116
	*p*	0.892	0.817	0.560	0.435	0.409	0.602	0.606	0.589	0.321	0.477
PANSS-T	*r*	−0.025	0.008	0.063	−0.12	−0.162	−0.118	0.137	−0.073	0.255	−0.124
	*p*	0.876	0.959	0.701	0.469	0.325	0.474	0.398	0.656	0.112	0.444
Venous pH	*r*	0.188	−0.061	−0.213	−0.091	−0.107	−0.069	−0.002	0.206	0.095	0.221
	*p*	0.244	0.707	0.186	0.581	0.517	0.676	0.991	0.203	0.561	0.171
PvO_2_	*r*	0.117	0.055	−0.056	0.147	0.148	0.081	0.030	0.272	−0.033	0.262
	*p*	0.472	0.737	0.732	0.373	0.368	0.625	0.855	0.089	0.838	0.102
PvCO_2_	*r*	−0.135	0.074	0.128	0.272	−0.052	−0.215	−0.010	−0.261	−0.112	−0.277
	*p*	0.406	0.648	0.432	0.094	0.753	0.189	0.952	0.104	0.49	0.084
Lactic acid	*r*	−0.052	−0.067	0.144	0.13	0.181	0.158	0.089	0.07	−0.184	0.079
	*p*	0.749	0.680	0.375	0.43	0.27	0.337	0.587	0.667	0.256	0.626
K^+^	*r*	−0.042	−0.232	−0.106	0.147	0.067	−0.008	0.010	0.068	0.116	0.068
	*p*	0.798	0.149	0.514	0.371	0.685	0.961	0.951	0.678	0.474	0.675
FBG	*r*	0.306	−0.127	0.098	−0.255	0.065	0.222	0.081	0.495	−0.028	0.515
	*p*	0.054	0.433	0.546	0.117	0.695	0.175	0.620	0.001^**^	0.862	0.001^**^
SOD	*r*	−0.248	−0.109	0.159	−0.124	0.268	0.395	−0.151	0.038	−0.017	0.075
	*p*	0.145	0.503	0.355	0.471	0.114	0.017^*^	0.380	0.828	0.920	0.662

*Pearson correlation*.

* *p < 0.05*,

** *p <0.01*.

**Table 4:** Correlation analysis between the general situation and the effect indicators in the control group.

		**Age**	**Education**	**Smoking history**	**Height**	**Weight**	**BMI**
Venous pH	*r*	0.336	−0.030	0.091	−0.229	0,020	0.205
	*p*	0.034^*^	0.865	0.577	0.166	0.977	0.217
PvO_2_	*r*	0.214	0.065	0.007	−0.179	−0.024	0.105
	*p*	0.185	0.691	0.968	0.283	0.885	0.530
PvCO_2_	*r*	−0.420	0.096	−0.090	0.423	0.136	−0.144
	*p*	0.007^**^	0.557	0.579	0.008^**^	0.415	0.389
Lactic acid	*r*	0.083	−0.124	−0.214	−0.021	−0.015	0.030
	*p*	0.611	0.445	0.186	0.901	0.927	0.858
K^+^	*r*	−0.014	−0.338	0.125	−0.020	0.016	0.042
	*p*	0.933	0.032^*^	0.443	0.904	0.923	0.802
FBG	*r*	0.157	−0.206	0.074	−0.143	−0.026	0.066
	*p*	0.333	0.203	0.651	0.391	0.878	0.694
SOD	*r*	−0.314	0.256	0.054	−0.016	−0.112	−0.166
	*p*	0.049^*^	0.110	0.742	0.925	0.503	0.318

*Pearson correlation*.

* *p < 0.05*,

** *p < 0.01*.

#### PANSS Scores and Other Effect Indicators

To explore the relationship between the PANSS scores and other variables, Pearson *r* was measured. It was found that K^+^ was negatively correlated with the PANSS-G and PANSS-T score, and SOD was negatively correlated with the PANSS-G score, whereas venous pH, PvO_2_, PvCO_2_, and lactic acid were no correlated with it (Table 5).

**Table 5:** Correlation analysis of PANSS scores and other effect indicators.

		**PANSS-P**	**PANSS-N**	**PANSS-G**	**PANSS-T**
Venous pH	*r*	0.185	0.039	0.209	0.195
	*p*	0.254	0.813	0.196	0.227
PvO_2_	*r*	−0.080	0.064	−0.003	0.002
	*p*	0.624	0.696	0.986	0.989
PvCO_2_	*r*	−0.081	−0.079	−0.231	−0.194
	*p*	0.619	0.628	0.151	0.230
Lactic acid	*r*	−0.093	0.162	−0.210	−0.077
	*p*	0.569	0.318	0.194	0.636
K^+^	*r*	−0.120	−0.233	−0.330	−0.332
	*p*	0.462	0.148	0.038^*^	0.036^*^
FBG	*r*	0.026	−0.167	0.068	−0.028
	*p*	0.874	0.302	0.677	0.864
SOD	*r*	−0.212	−0.059	−0.357	−0.298
	*p*	0.190	0.720	0.024^*^	0.062

*Pearson correlation*.

* *p < 0.05*.

#### Venous pH, PvO_2_, PvCO_2_, and Other Effect Indicators

To explore the relationship between venous pH, PvO_2_, PvCO_2_, and other variables, Pearson *r* was measured both in the SCH group and the control group. It was found that K^+^ was negatively correlated with PvO_2_, and lactic acid was negatively correlated with venous pH in the SCH group (Table 6), whereas FBG was positively correlated with venous pH and PvO_2_ negatively correlated with PvCO_2_, and lactic acid was positively correlated with PvCO_2_ and negatively correlated with venous pH in the control group (Table 7).

**Table 6:** Correlation analysis of venous pH, PvO_2_, PvCO_2_, and other effect indicators in the SCH group.

		**Venous pH**	**PvO_**2**_**	**PvCO_**2**_**
K^+^	*r*	−0.259	−0.184	−0.327
	*p*	0.106	0.256	0.040^*^
FBG	*r*	0.222	−0.059	−0.273
	*p*	0.168	0.720	0.088
Lactic acid	*r*	−0.367	−0.218	0.260
	*p*	0.020^*^	0.177	0.106
SOD	*r*	−0.112	0.105	−0.164
	*p*	0.491	0.518	0.311

*Pearson correlation*.

* *p < 0.05*.

**Table 7:** Correlation analysis of venous pH, PvO_2_, PvCO_2_, and other effect indicators in the control group.

		**Venous pH**	**PvO_**2**_**	**PvCO_**2**_**
K^+^	*r*	0.249	−0.080	−0.184
	*p*	0.121	0.622	0.254
FBG	*r*	0.701	0.357	−0.595
	*p*	<0.001^*^	0.024^**^	<0.001^*^
Lactic acid	*r*	−0.739	−0.302	0.503
	*p*	<0.001^*^	0.058	0.001^*^
SOD	*r*	−0.036	−0.079	−0.007
	*p*	0.827	0.628	0.963

*Pearson correlation*.

* *p < 0.01*,

** *p < 0.05*.

### Multiple Linear Regression Analysis of PvO_2_

To explore the relationship between PvO_2_ and other variables, Pearson *r* was measured.

In acute SCH patients, it was found that PvCO_2_ exhibited negative correlation with PvO_2_ (*r* = −0.581, *p* < 0.001), whereas venous pH value showed positive correlation with it (*r* = 0.650, *p* < 0.001). Based on these results, a multiple linear regression analysis was performed with venous pH and PvCO_2_ as the independent variables and PvO_2_ as the dependent variable. As a result, venous pH was the only variable remaining in the regression model (*t* = 5.268, *p* < 0.001, unstandardized coefficient = 880.699, standardized coefficient = 0.650, constant = −6,420.858). The model was tested with *F*_(1, 38)_ = 27.747, *p* < 0.001, indicating that the venous pH was significant. Thus, the regression model could be described as follows: PvO_2_ = −6,420.858 + 880.699 venous pH. The absolute values of the standardized residual and the student-adjusted residual were <3.

In stable SCH patients, it was found that PvCO_2_ exhibited negative correlation with PvO_2_ (*r* = −0.784, *p* < 0.001), whereas venous pH value showed positive correlation with it (*r* = 0.655, *p* < 0.001). Venous pH was still the only variable remaining in the regression model (*t* = 4.663, *p* < 0.001, unstandardized coefficient = 568.689, standardized coefficient = 0.655, constant = −4,124.051). The model was tested with *F*_(1, 30)_ = 21.739, *p* < 0.001, indicating the venous pH was significant.

In the control group, it was found that PvCO_2_ exhibited negative correlation with PvO_2_ (*r* = −0.672, *p* < 0.001), whereas venous pH value showed positive correlation with it (*r* = 0.575, *p* < 0.001). PvCO_2_ was the only variable remaining in the regression model (*t* = −5,594, *p* < 0.001, unstandardized coefficient = −0.654, standardized coefficient = −0.672, constant = 68.118). The model was tested with *F*_(1, 38)_ = 31.291, *p* < 0.001, indicating that the PvCO_2_ was significant.

### Comparison of Effect Indexes in Acute and Stable SCH Patients

Only 31 patients finished the evaluation after pharmacotherapy, and a paired-samples *t*-test was performed between before and after treatment in the SCH group. The results of each effect index were compared before and after treatment, The scores of PANSS and subscales were decreased, with the reduction rates at 48.82% (PANSS-P), 28.92% (PANSS-N), 28.07% (PANSS-G), and 34.39% (PANSS-T), respectively. This indicated that the patients were in a state of stabilization.

The venous pH value was also decreased after treatment (*p* < 0.05), whereas K^+^ was increased after treatment (*p* < 0.01). Other effect indexes showed no significant difference in acute and stable SCH patients, whereas the *p*-values of PvO_2_ (0.061) and PvCO_2_ (0.056) in the *t*-test were close to the critical value of 0.05 (Table 8).

**Table 8:** Comparison of effect indexes in acute and stable SCH patients (mean ± SD).

**Variable**	**Acute**	**Stable**	***t***	***P***
	***n* = 31**	***n* = 31**		
PANSS-P	24.58 ± 5.08	12.84 ± 4.01	11.569	<0.001^*^
PANSS-N	18.29 ± 7.57	13.00 ± 4.55	5.640	<0.001^*^
PANSS-G	36.23 ± 7.15	26.06 ± 4.28	9.730	<0.001^*^
PANSS-T	79.10 ± 15.41	51.90 ± 9.84	11.566	<0.001^*^
Venous pH	7.373 ± 0.030	7.357 ± 0.037	2.430	0.021^**^
PvO_2_ (mm Hg)	74.58 ± 41.96	60.03 ± 32.12	1.949	0.061
PvCO_2_ (mm Hg)	46.03 ± 6.67	48.45 ± 8.53	−1.992	0.056
Lactic acid (mol/L)	2.17 ± 1.17	2.28 ± 0.65	−0.526	0.503
K^+^ (mol/L)	3.72 ± 0.35	3.99 ± 0.35	−3.511	0.001^*^
FBG (mol/L)	5.13 ± 0.94	5.30 ± 1.30	−0.612	0.545
SOD (U/ml)	157.19 ± 34.04	164.90 ± 21.47	−1.292	0.206

*Paired-samples t-test*.

* *p < 0.01*,

** *p < 0.05*.

To determine whether VBG analysis and SOD results are trait or state variables in schizophrenia, the stable period outcomes of these indicators were compared with healthy controls. It was shown that the venous pH, PvO_2_, K^+^, and FBG values in the stable period were still higher than those in healthy controls, although it is lower than that in the acute period, whereas PvCO_2_, lactic acid, and SOD levels were lower, indicating statistical significance (*p* < 0.001 in all) (Table 9).

**Table 9:** Comparison of effect indexes between stable SCH patients and control group (mean ± SD).

**Variable**	**SCH (stable)**	**Control group**	***t***	***P***
	***n* = 31**	***n* = 40**		
Venous pH	7.357 ± 0.037	7.284 ± 0.051	6.722	<0.001^*^
PvO_2_ (mm Hg)	60.03 ± 32.12	30.00 ± 10.72	5.539	<0.001^*^
PvCO_2_ (mm Hg)	48.45 ± 8.53	58.33 ± 11.02	−4.254	<0.001^*^
Lactic acid (mol/L)	2.28 ± 0.65	3.55 ± 1.27	−5.027	<0.001^*^
K^+^ (mol/L)	3.99 ± 0.35	3.54 ± 0.25	6.228	<0.001^*^
FBG (mol/L)	5.30 ± 1.30	4.05 ± 0.93	4.728	<0.001^*^
SOD (U/mL)	164.90 ± 21.47	196.38 ± 14.85	−6.972	<0.001^*^

*Independent-samples t-test*.

* *p < 0.01*.

## Discussion

### Abnormal SOD in SCH

Since the role for toxic radicals in the etiology of schizophrenia was proposed in 1950s (19), there have been more and more investigations of free radical metabolism in schizophrenia. To protect biological systems from free radical toxicity, several cellular antioxidant defense mechanisms keep the production of ROS in check, including enzymatic and non-enzymatic pathways (20). The primary antioxidant enzymes against superoxide radicals include SOD, catalase (CAT), and glutathione peroxidase. To prevent lipid peroxidation by superoxide and hydroxyl radicals, superoxide is first converted by SOD to hydrogen peroxide, which is then decomposed to water and oxygen by CAT, thereby preventing the formation of hydroxyl radicals (21).

SOD is one of the critical scavenging enzymes that have been reported most commonly in schizophrenia. There are different results about SOD in SCH. Some researchers have reported an increase in SOD activity in the blood of SCH patients (5, 6, 22–24), whereas others have reported reduced SOD activity in neuroleptic-naive first-episode schizophreniform and schizophrenic patients (25–27). Decreased levels of antioxidant enzymes may have a direct implication of the oxidative stress; the increased levels may reflect a compensatory effect or a preceding oxidative stress in the cell. The difference in the direction of the changes may be attributed not only to some confounders such as clinical symptoms and therapeutic features, but also to the dynamic status of the antioxidant enzymes, which have an intricate balance with other biological pathways and systems (28).

According to the meta-analysis results, the level of activity of erythrocyte SOD was reduced in acuter elapse of psychosis, drug-naive first-episode psychosis, stable medicated outpatients, and chronic inpatients (7). These suggest that antioxidant defenses are reduced in schizophrenia. In our study, we found that the level of SOD in the SCH group was lower than that in the control group (*p* < 0.001) not only in the acute episode, but also in the stable stage. Moreover, in the acute phase, SOD activity was negatively correlated with the general pathological score of PANSS. It suggests that there is abnormal oxidative stress (decreased antioxidant defense) in schizophrenia and that acute psychotic symptoms are inversely correlated with antioxidant capacity.

### Histogenous Hypoxia and SCH

Although there is much indirect evidence linking schizophrenia to hypoxia (11–13), but the direct evidence of hypoxia in SCH has never been found up to now. In our study, the SCH patients exhibited higher PvO_2_ levels and lower PvCO_2_ levels compared with the control group, which suggests that the SCH patients experience significant differences in oxygen (O_2_) utilization.

The continuous exchange of material between circulating blood and tissue fluids is vital; diffusion, which is the primary process facilitating the exchange of O_2_ and carbon dioxide (CO_2_), occurs due to the difference in concentration of the substance undergoing diffusion across either side of the blood vessel wall. When arterial blood flows through capillaries and the partial pressure of O_2_ in the blood is higher than that in the tissue fluid, the O_2_ will spread into the tissue fluid. The partial pressure of CO_2_ in tissue fluid, however, is higher than that in blood; therefore, diffusion of CO_2_ into the blood occurs. The diffusion rate is positively correlated to the partial pressure difference between the arterial and venous O_2_ and CO_2_. The difference in arterial and venous O_2_ partial pressure may reflect the conditions under which O_2_ release, O_2_ uptake, and the utilization of O_2_ in tissues take place, and therefore, it can be used to study blood circulation and metabolism in tissues (29).

Arterial blood gas analysis was not undertaken as part of the present study. Even discounting the differences in partial pressure of arterial oxygen (PaO_2_) levels and taking only into consideration the decreased PvO_2_ results found in the present study, it can be demonstrated that histogenous hypoxia exists in relation to SCH, because these are symptoms of histogenous hypoxia (30).

### Acid Retention and SCH

The final metabolites of carbohydrates are pyruvic acid, CO_2_, and H_2_O, whereas lipids are broken down into fatty acids and glycerol, with the latter providing energy through glycolysis or aerobic oxidation. Under the condition of sufficient O_2_ supply, fatty acids can be oxidized to decompose into CO_2_ and H_2_O. Proteins are broken down into amino acids, and the final metabolites are ammonia and α-ketonic acid (31). Accordingly, most of the final metabolites of the three major nutrients in the human body are acidic; furthermore, the metabolic wastes discharged by the body, such as exhaled CO_2_, sweat, and urine, are also acidic (32). That is, the human body is an organism that continuously produces and discharges acids.

As arterial blood flows through tissues and cells, it releases oxygen and nutrients, takes away carbon dioxide and metabolic wastes produced by tissues and cells, and forms venous blood. The relationship between the human body and venous blood was similar to the relationship between the city and sewer. For a city of the same size, the more garbage and waste there are in the sewer, the cleaner the city will be. Conversely, if the sewers are cleaner, the dirtier the city is, and the more waste that is left behind. Likewise, for a body continuously producing acid, the more acidic the venous blood is—indicating that more waste products are discharged—the healthier the body is.

In our study, the SCH patients exhibited higher venous pH levels and lower PvCO_2_/lactic acid levels compared with the control group. These results indicate that the SCH patients have significantly reduced acid excretion from tissue cells, compared with the healthy controls. Thus, acid retention exists in relation to SCH.

### Histogenous Hypoxia and Acid Retention

The increased PvO_2_ is closely related to an increase in venous pH in acute and stable SCH patients, and the increased PvO_2_ is closely related to a decrease in PvCO_2_ in the control group, which was demonstrated through multiple linear regression analyses in the current research. The present study's authors found the same result in previous research concerning depression (14). The reason for this result may relate to the influences of the pH and PvCO_2_ of venous blood on the O_2_ dissociation curve, which will move to the right in conditions comprising decreased pH or increased PvCO_2_, with decreased affinity between hemoglobin and O_2_, or will move to the left in conditions of increased pH or decreased PvCO_2_, with increased affinity between hemoglobin and O_2_ (32). The venous blood pH value is significantly higher, and PvCO_2_ is significantly lower in SCH patients than in participants in the healthy control group. Thus, the O_2_ dissociation curve will move to the left, and therefore the affinity between hemoglobin and O_2_ will increase, and O_2_ release is impaired (30). This may offer some explanation toward the tendency for histogenous hypoxia in SCH.

### The Effect of Pharmacotherapy to Histogenous Hypoxia and Acid Retention

In this study, SOD activity showed no significant difference in acute and stable period. PvO_2_ showed a downward trend after treatment. It suggests that there was a downward trend in histogenous hypoxia after treatment.

The venous pH value was decreased significantly after combination treatment of AAS and Chinese medicine decoction together with the improvement of mental symptoms. It was indicated that the acid retention in tissues and cells was improved. In traditional Chinese medicine, phlegm and stasis are very important pathogenesis of schizophrenia (18, 33). According to clinical observation, the endogenous turbid dampness induced by disorder of spleen transportation will block the movement of Qi and blood and cause Qi stagnation or blood stasis, which is equivalent to the metabolic disorder in modern medicine (34). Ditan Huayu Lishen decoction might remove metabolic wastes in the body and promote the excretion of acid metabolites by removing phlegm and blood stasis. On the other hand, psychiatric symptoms (such as day–night reversal, eating irregularity, impulsive behavior) can be relieved by antipsychotic drugs, so the patients would live a regular life and reduce the generation of acid metabolites.

Although the venous pH improved significantly, both the venous pH and PvO_2_ were higher, and PvCO_2_ was lower in acute and stable SCH patients than that in healthy controls. Thus, venous pH, PvO_2_, and PvCO_2_ are trait variables, but not state variables of SCH. In the authors' previous study, abnormalities in VBG analysis were also found in depression. The venous pH, PvO_2_, and PvCO_2_ were 7.368 ± 0.026 (Cohen *d* = 1.079), 32.523 ± 11.518 mm Hg (Cohen *d* = 1.099), and 46.635 ± 5.708 mm Hg (Cohen *d* = −0.806) respectively (14). It showed higher venous pH, PvO_2_, and lower PvCO_2_ in SCH than that in depression and larger effect size in SCH than that in depression. It seems that diseases with greater acid retention/histogenous hypoxia may be more difficult to treat, because it is clear that schizophrenia is associated with greater acid retention and histogenous hypoxia than depression.

### The Role of VBG in Pathophysiology for SCH

In our study, the FBG showed no difference in acute and stable SCH patients, but they were all higher than that in the control group. This suggests abnormalities in glucose metabolism in patients with SCH that are unaffected by short-term drug therapy. The FBG was positively correlated with venous pH and PvO_2_ and negatively correlated with PvCO_2_ in the control group; this suggests that glucose metabolism is closely related to hypoxia and acid retention.

Ryan et al. found that inpatients with first-episode schizophrenia who had never taken medication had significantly impaired glucose tolerance and elevated insulin levels compared with controls (35). Spelman et al. found that 10.5% of untreated hospitalized patients had impaired glucose tolerance and 18.2% of their relatives in Irish, whereas healthy controls had no impaired glucose tolerance (36). In a meta-analysis about FBG levels in first-episode antipsychotic-naive individuals with first-episode schizophrenia, it was also found that glucose homeostasis was altered from illness onset in schizophrenia, indicating that patients were at increased risk of diabetes as a result (37). The possible reason of these is that acid retention and hypoxia exist in schizophrenia, and blood glucose is affected by acid retention and hypoxia.

Recently, the decrease in pH value and the increase in lactic acid level in the brain of schizophrenia have become a hot research topic. Significantly lower pH and higher lactate levels in the brains relative to controls, as well as a significant negative correlation between pH and lactate levels in a meta-analysis about SCH model mice, were observed (38). Another meta-analysis of six postmortem studies revealed a significant increase in lactate, but found a non-significant decrease in pH in schizophrenia brain (39). The authors also explored the underlying mechanisms of lactic acid increase and pH decrease in the brain of schizophrenia: mitochondrial dysfunction, oxidative stress, and hypoxia-related changes in gene expression lead to downregulation of the TCA cycle and oxidative phosphorylation and to an increased reliance on glycolysis for energy production (39).

The findings of this study may explain the above phenomenon. Acid retention and tissue hypoxia were found in schizophrenia. Because of acid retention, less acid is excreted from venous blood (increased pH and decreased lactic acid), resulting in a decrease in pH and increase in lactic acid in the tissues and cells of the body. Glucose metabolism is closely related to hypoxia and acid retention, which will lead to mitochondrial dysfunction and energy metabolism disorders. There are several evidences to suggest that altered energy metabolism is present even at the earliest stages of SCH and may, in fact, contribute to the development of SCH (39). The abnormal glucose metabolism found in SCH in this study is one of the manifestations of energy metabolism disorder. Thus, histogenous hypoxia and acid retention may play an important role in pathophysiology for SCH.

### Limitation

One of the limitations of this study is that only the combination of traditional Chinese medicine and Western medicine was used as the intervention method, and the control group of pure Western medicine was absent. Therefore, the effect produced by the combination drug therapy could not be distinguished from the effect of Western medicine or Chinese medicine. It needs to be further improved in future research.

Another limitation is the large difference in educational attainment between the SCH group and the healthy control group. Differences in educational attainment may imply differences in socioeconomic status, nutritional status, and physical activity. Although there was no significant correlation between effect indicators and education years in correlation analysis subsequently, the impact of this difference on the overall results still needs to be concerned.

## Conclusion

In this study, we compared results pertaining to VBG analysis and SOD of SCH patients vs. healthy participants in a control group. We found higher venous pH, PvO_2_, and FBG levels and lower SOD, lactic acid, and PvCO_2_ levels in SCH patients compared with the control group. Based on these findings, we inferred that histogenous hypoxia and acid retention exist in relation to SCH. We also compared results pertaining to VBG analysis and SOD of acute and stable SCH patients. We found that SOD activity showed no significant difference between acute and stable period, PvO_2_ showed a downward trend, and venous pH was decreased significantly in the stable period. We inferred that acid retention in tissues and cells was improved.

Both the venous pH and PvO_2_ were higher and PvCO_2_ was lower in acute and stable SCH patients than those in healthy controls. Thus, venous pH, PvO_2_, and PvCO_2_ are trait variables, but not state variables of SCH.

The theory of histogenous hypoxia and acid retention can well explain the decrease in pH value and the increase in lactic acid in brain tissue of patients with schizophrenia. Histogenous hypoxia and acid retention also affect glucose metabolism and cause energy metabolism disorder. Thus, they may play an important role in pathophysiology for SCH.

## Data Availability Statement

The original contributions presented in the study are included in the article/supplementary material, further inquiries can be directed to the corresponding author/s.

## Ethics Statement

The studies involving human participants were reviewed and approved by the Ethics Committee of Affiliated Brain Hospital of Guangzhou Medical University. The patients/participants provided their written informed consent to participate in this study.

## Author Contributions

H-YH designed the study. Q-LL and XH enrolled patients into the study and contributed to data collection. YL assisted with data collection. X-MZ and Y-BZ finished the lab tests. Q-LL did the statistical analysis and H-YH supervised it. XH and H-YH interpreted the data. XH wrote the original draft of the paper and H-YH revised it. All authors contributed to the article and approved the submitted version.

## Funding

This research was funded by the Guangdong Province Administration of Traditional Chinese Medicine (Program Number: 20171209), Affiliated Brain Hospital of Guangzhou Medical University.

## Conflict of Interest

The authors declare that the research was conducted in the absence of any commercial or financial relationships that could be construed as a potential conflict of interest.

## Publisher's Note

All claims expressed in this article are solely those of the authors and do not necessarily represent those of their affiliated organizations, or those of the publisher, the editors and the reviewers. Any product that may be evaluated in this article, or claim that may be made by its manufacturer, is not guaranteed or endorsed by the publisher.

## Abbreviations

FBG: fasting blood glucose
K^+^: potassium ion
PANSS: Positive and Negative Syndrome Scale
PANSS-G: general psychopathology subscale of PANSS
PANSS-N: negative subscale of PANSS
PANSS-P: positive subscale of PANSS
PANSS-T: total score of PANSS
PvCO_2_: partial pressure of venous carbon dioxide
PvO_2_: partial pressure of venous oxygen
SCH: schizophrenia
SOD: superoxide dismutase
VBG: venous blood gas.
